# Monitoring Expired CO_2_ Kinetics to Individualize Lung-Protective Ventilation in Patients With the Acute Respiratory Distress Syndrome

**DOI:** 10.3389/fphys.2021.785014

**Published:** 2021-12-21

**Authors:** Fernando Suárez-Sipmann, Jesús Villar, Carlos Ferrando, Juan A. Sánchez-Giralt, Gerardo Tusman

**Affiliations:** ^1^CIBER de Enfermedades Respiratorias, Instituto de Salud Carlos III, Madrid, Spain; ^2^Intensive Care Unit, Hospital Universitario La Princesa, Madrid, Spain; ^3^Department of Surgical Sciences, Anesthesiology & Critical Care, Hedenstierna Laboratory, Uppsala University Hospital, Uppsala, Sweden; ^4^Multidisciplinary Organ Dysfunction Evaluation Research Network (MODERN), Research Unit, Hospital Universitario Dr. Negrín, Las Palmas de Gran Canaria, Spain; ^5^Keenan Research Center at the Li Ka Shing Knowledge Institute, St. Michael’s Hospital, Toronto, ON, Canada; ^6^Department of Anesthesiology and Critical Care, Hospital Clinic, Barcelona, Spain; ^7^Hospital Clinic, Institut d’Investigacions Biomèdiques August Pi i Sunyer (IDIBAPS), Barcelona, Spain; ^8^Department of Anesthesiology, Hospital Privado de Comunidad, Mar del Plata, Argentina

**Keywords:** volumetric capnography, dead space, acute respiratory distress syndrome, ventilator-induced lung injury, mechanical ventilation

## Abstract

Mechanical ventilation (MV) is a lifesaving supportive intervention in the management of acute respiratory distress syndrome (ARDS), buying time while the primary precipitating cause is being corrected. However, MV can contribute to a worsening of the primary lung injury, known as ventilation-induced lung injury (VILI), which could have an important impact on outcome. The ARDS lung is characterized by diffuse and heterogeneous lung damage and is particularly prone to suffer the consequences of an excessive mechanical stress imposed by higher airway pressures and volumes during MV. Of major concern is cyclic overdistension, affecting those lung segments receiving a proportionally higher tidal volume in an overall reduced lung volume. Theoretically, healthier lung regions are submitted to a larger stress and cyclic deformation and thus at high risk for developing VILI. Clinicians have difficulties in detecting VILI, particularly cyclic overdistension at the bedside, since routine monitoring of gas exchange and lung mechanics are relatively insensitive to this mechanism of VILI. Expired CO_2_ kinetics integrates relevant pathophysiological information of high interest for monitoring. CO_2_ is produced by cell metabolism in large daily quantities. After diffusing to tissue capillaries, CO_2_ is transported first by the venous and then by pulmonary circulation to the lung. Thereafter diffusing from capillaries to lung alveoli, it is finally convectively transported by lung ventilation for its elimination to the atmosphere. Modern readily clinically available sensor technology integrates information related to pulmonary ventilation, perfusion, and gas exchange from the single analysis of expired CO_2_ kinetics measured at the airway opening. Current volumetric capnography (VCap), the representation of the volume of expired CO_2_ in one single breath, informs about pulmonary perfusion, end-expiratory lung volume, dead space, and pulmonary ventilation inhomogeneities, all intimately related to cyclic overdistension during MV. Additionally, the recently described capnodynamic method provides the possibility to continuously measure the end-expiratory lung volume and effective pulmonary blood flow. All this information is accessed non-invasively and breath-by-breath helping clinicians to personalize ventilatory settings at the bedside and minimize overdistension and cyclic deformation of lung tissue.

## Introduction

The acute respiratory distress syndrome (ARDS) is the most severe form of acute respiratory failure that affects the lungs in a heterogeneous way, profoundly impairing their mechanical properties and gas-exchange functions. Diffuse lung pan-endothelial inflammation, the hallmark of the syndrome, leads to the invasion of alveolar spaces by edema and inflammation reducing effective pulmonary lung volume (i.e., functional residual capacity, FRC). The lung becomes heavier exerting a superimposed pressure on the dependent parts of the lung, critically decreasing regional transpulmonary pressure. This further reduces FRC by promoting lung collapse, a common pathophysiological feature of ARDS (Gattinoni et al., 2006).

Mechanical ventilation (MV) is the principal life-support intervention in the management of patients with ARDS but at the risk to perpetuate or aggravate lung damage. Ventilation-induced lung injury (VILI) results from the need to use high transpulmonary pressures and frequently higher tidal volumes (VT) to oxygenate and ventilate the heterogeneously ARDS lung. The delivered VT is distributed in a much smaller lung volume with important regional differences, with three important consequences: (1) less diseased lung regions will receive a larger fraction of the VT causing an increased cyclic tissue deformation or strain, a mechanism that triggers lung inflammation, (2) more diseased but ventilated regions will be submitted to a higher transpulmonary pressure for any given delivered VT increasing the local mechanical stress also known as cyclic overdistension, and (3) collapsed regions eventually re-open and close with each tidal inflation and deflation causing cyclic recruitment–de-recruitment, which can be considered an extreme form of regional strain. These are three of the most important mechanisms by which MV damages lung parenchyma. Lung imaging techniques, such as CT or intravital microscopy, have confirmed the heterogeneity of lung injury in ARDS at different scales, revealing the coexistence of normal, collapsed, and overdistended alveoli in different lungs regions. The resulting non-uniform distribution of tidal ventilation can be visualized in real time at the bedside by electrical impedance tomography (EIT), whereas more sophisticated techniques, such as PET and single-photon emission tomography (SPECT) imaging, have located the main inflammatory response in normally ventilated areas but not in collapsed ones (Bellani et al., 2009, 2011). Thus, lung collapse acts as a stress-raiser since it contributes to lung heterogeneity creating areas receiving an excessive VT in relation to their regional volume.

The introduction of lung-protective ventilation strategies aimed at reducing the mechanical stress imposed by the ventilator has contributed to reducing morbidity and mortality of patients with ARDS (ARDS Network, 2000). Ideally, these strategies should be individualized, but this requires useful and directed bedside clinical monitoring. However, routine monitoring only includes basic lung mechanics, gas exchange and intermittently, lung imaging techniques and thus, the clinical detection of overdistension or lung strain, and VILI remains difficult. The analysis of expired gases, in particular CO_2_, is a well-established, robust, and clinically accessible monitoring option. Volumetric capnography (VCap), representing the volume of CO_2_ expired in one breath, has specific features regarding the analysis of body CO_2_ kinetics that can be of great value in detecting lung overdistension by providing continuous non-invasive information on lung perfusion, convective gas transport, lung diffusion, and dead space ventilation (VD), all intimately related and sensitive to the effects of lung overdistension. However, this source of highly relevant biological information is still largely underused in clinical practice. A progressive better understanding of the complex behavior and physiology of CO_2_ kinetics have led to recent new developments for advanced analysis of the volumetric capnogram and a derived capnodynamic method that hold great promise in overcoming the difficulties in adopting the expired CO_2_ monitoring in routine clinical practice.

The aim of this manuscript is to review the principles, uses, and physiological basis of expired CO_2_ kinetics monitoring. We will review new developments and value of VD monitoring using VCap in patients with ARDS and describe the new capnodynamic method that continuously monitors effective lung volume and perfusion in a non-invasive way.

## Volumetric Capnography

**FIGURE 1:**
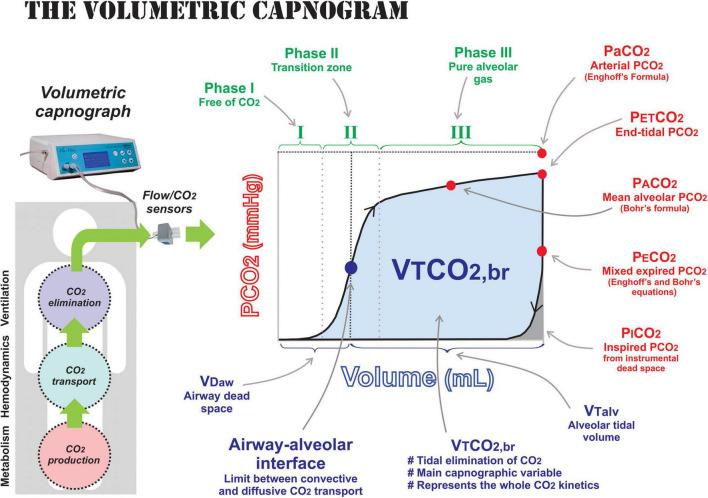
Components of volumetric capnography. The volumetric capnogram is the plot of expired CO_2_ in one tidal breath measured by mainstream flow and CO_2_ sensors. The capnogram phases are shown in green: phase I is the portion of the expired gas without CO_2_ that represents pure airway dead space; phase II is the transition zone composed by the progressive emptying of lung units with different time-constants and spatial distribution; and phase III is the pure alveolar gas expired once airway dead space (VD_aw_) has been washed out. The relevant partial pressures of CO_2_ measured by VCap are presented in red: end-tidal (PETCO_2_), mean alveolar (PACO_2_), mixed expired (P$\bar{\text{E}}$CO_2_), and inspired (PICO_2_) pressure. The arterial PaCO_2_ is not directly measured, but it included for referencing of the other partial pressures. The volumes obtained by the VCap are presented in blue: volume of airway dead space (VD_aw_), alveolar tidal volume (VT_alv_), and volume of CO_2_ eliminated per breath or area under the VCap curve (VTCO_2,br_). The blue dot represents the airway–alveolar interface, the limit between convective and diffusive CO_2_ transport close to the entrance of lung acini and, therefore, it also morphologically separates the conducting from the gas-exchanging lung compartment.

The integration of an infrared CO_2_ sensor and a flow sensor in a mainstream configuration allows for the reconstruction of the volumetric capnogram at the airway opening (Figure 1). As the cardiorespiratory system has an open arrangement, VCap contains implicit information regarding these systems expressed not only in their derived variables and indexes but also in its shape that helps the interpretation of normality and diseases throughout its derived-parameters. As mentioned above, CO_2_ kinetics is context-sensitive, which means that VCap parameters must be interpreted when changes in metabolism, pulmonary perfusion, or ventilation occur one at a time (Sipmann et al., 2014). For example, an increase in VT improves CO_2_ elimination explained solely by a ventilatory change when metabolism and hemodynamics are constant.

Interpretation of CO_2_ kinetics can be done during steady and non-steady state conditions, although the non-steady state is of particular interest in clinical monitoring as it allows to interpret and react to changes in pulmonary perfusion, end-expiratory lung volume, and/or alveolar ventilation after adjusting the ventilatory settings or to changing clinical conditions.

## Rationale for the Measurement of Dead Space and Alveolar Co_2_ by Volumetric Capnography

**FIGURE 2:**
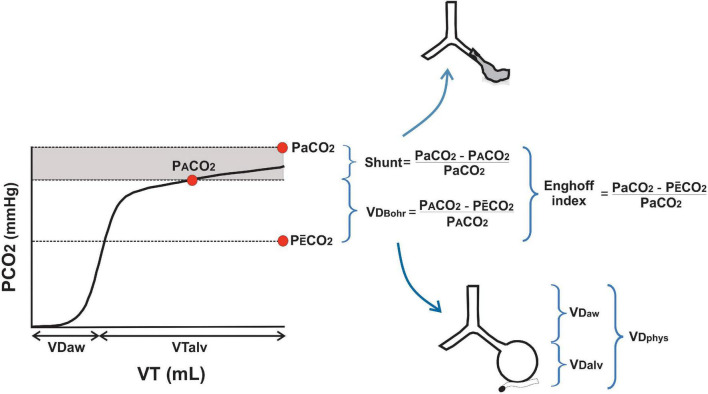
Differences between Bohr’s and Enghoff’s approaches. The main physiological difference between Bohr’s dead space (VD_Bohr_) and Enghoff’s index is visualized in one volumetric capnogram. While VD_Bohr_ represents true dead space in non-perfused alveoli and main conducted airways, the Enghoff’s index includes also the effect of shunt and low V/Q in its calculation (gray area). PaCO_2_, PACO_2_, and P$\bar{\text{E}}$CO_2_ are the arterial, alveolar, and mixed expired partial pressure of carbon dioxide, respectively. VD_phys_, VD_aw_, and VD_alv_ are physiological, airway, and alveolar dead spaces, respectively. VT is the tidal volume and VT_alv_ is the alveolar tidal volume.

Dead space ventilation is the wasted portion of ventilation not involved in gas exchange *per se*. It constitutes the evolutionary adaptive price paid by all mammals that depend on the bulk convective transport of ambient air to the gas–liquid gas-exchange interface, the alveolar–capillary membrane. By the end of the 19th century, Christian Bohr presented a method to estimate VD volume based on the mass balance of any gas breathed during the respiratory cycle (Figure 2) (Bohr, 1891). He brilliantly adapted his formula using CO_2_ as the tracer gas, which still constitutes the basis of the clinical measurement of dead space. Aitken and Clark-Kennedy (1928) were the first to propose to represent CO_2_ in relation to the expired volume, the VCap. Since then, the usefulness of this tool to calculate VD has been highlighted by many authors. Nowadays, major advances in CO_2_ and flow sensing technology together with powerful hardware and computation capabilities have made real-time breath-by-breath VD calculations possible. The key feature to calculate VD using VCap relies on the precise measurement of mixed expired (P$\bar{\text{E}}$CO_2_) and the mean alveolar (PACO_2_) partial pressures of CO_2_. The first necessary step was the simplification of the measurement of P$\bar{\text{E}}$CO_2_, which represents the dilution of CO_2_ within the lungs caused by the dead space effect. Initially, it could only be obtained by the cumbersome measurement using the Douglas’ bag. Currently, P$\bar{\text{E}}$CO_2_ can be measured by VCap in one single breath by calculating the mixed expired fraction of CO_2_ (F$\bar{\text{E}}$CO_2_), which is then transformed to partial pressure and expressed in BTPS as follows:


$$F\,\bar{\text{E}}\,{\mathrm{CO}}_{2}={\text{VTCO}}_{2,\mathrm{br}}/\text{VT}$$

$$P\,\bar{\text{E}}\,{\mathrm{CO}}_{2}=F\,\bar{\text{E}}\,{\mathrm{CO}}_{2}\times \left(\text{BP}-{\text{PH}}_{2}\,\text{O}\right)$$

where VTCO_2,br_ is the amount of CO_2_ expired in one VT, P is barometric pressure, and PH_2_O is water vapor pressure. The measurement of P$\bar{\text{E}}$CO_2_ by VCap has been validated by different research groups, all showing good agreement with the Douglas bag method (Lum et al., 1998; Sinha and Soni, 2012) or a metabolic monitor (Kallet et al., 2005; Siobal et al., 2013) in children (Lum et al., 1998) and adults (Sinha and Soni, 2012) also with ARDS (Kallet et al., 2005; Siobal et al., 2013). More recently, using a more advanced analysis of VCap, Doorduin et al. (2016) compared P$\bar{\text{E}}$CO_2_ measured by VCap versus the Douglas’ bag in patients with ARDS and found a bias of 0.2 mmHg and limits of agreement of − 3.0 to 4.5 mmHg (Doorduin et al., 2016). Our data in an animal model of ARDS using the multiple inert gas elimination technique (MIGET), the gold standard method for gas-exchange analysis supported the above findings. Our group found a close correlation of P$\bar{\text{E}}$CO_2_ measured by VCap with MIGET (*r* = 0.92; *p* < 0.0001), with a mean bias of −0.5 mmHg and limits of agreement between −2.5 and 1.5 mmHg (Tusman et al., 2011a).

The second, more recently introduced step, was the possibility to measure the mean alveolar partial pressure of CO_2_ (PACO_2_). This another essential component of the Bohr’s formula is difficult to measure and is still controversial parameter because PACO_2_ varies topographically and temporarily within inhomogeneous lungs along the respiratory cycle, even in healthy patients. This means that any single lung unit has its own PACO_2_ according to its respective V/Q ratio. Therefore, many controversies arose about what the “alveolar gas” really means and what is the proper definition and representative value of PACO_2_ (Rossier and Buhlmann, 1955). Two different approaches to describe the alveolar gas have been proposed in the past: the *ideal* and the *expired* alveolar gas. The *ideal alveolar gas* concept described by Riley and Cournand is based on the convenient and didactic assumption that the lung behaves as a perfect unit, where PACO_2_ equals capillary PCO_2_ (Riley and Cournand, 1949). However, this condition does not really exist even in healthy subjects due to the presence of airway dead space, anatomical shunt, stratified inhomogeneities, spatial and temporal V/Q mismatches, and incomplete gas mixing of inspired gases within the lungs (Fletcher et al., 1981; Crawford et al., 1985; Verbanck and Paiva, 2013). The impossibility to estimate PACO_2_ in the past was solved by Henrik Enghoff who suggested to use PCO_2_ in arterial blood (PaCO_2_) in the Bohr’s formula as a surrogate of PACO_2_ (Enghoff, 1938). This solution to calculate VD is still used today although, strictly speaking, it calculates not only dead space but also all the spectrum of V/Q mismatch present in the lung (Figure 2). The *expired alveolar air* concept offers a better and more realistic approximation to PACO_2_. Alveolar CO_2_ fluctuates during the respiratory cycle changing ∼4 mmHg between inspiration and expiration. DuBois described the “mean” PACO_2_ as the absolute value representing the whole lung (Dubois et al., 1952). This concept was confirmed decades later using complex mathematical models that included other aspects that affect alveolar gas composition such as pulmonary capillary pulsatile blood flow, capillary recruitment, solubility of CO_2_ in pulmonary tissue, and CO_2_ chemical reactions in blood (Hlastala, 1972). The alveolar gas must necessarily be measured during expiration due to the location of the CO_2_ sensor at the airway opening where inspiratory gases washes-out any remanent CO_2_. Mean PACO_2_ can conveniently be found at the midpoint of the phase III of the VCap. This phase is exclusively composed of alveolar gas where its slope represents the emptying of CO_2_ from all alveoli at different rates during the expiratory time (Fletcher et al., 1981; Tusman et al., 2012). We tested this hypothesis in an animal model of ARDS by comparing the VCap-based PACO_2_ with the one derived from the alveolar gas equation solved with data obtained from MIGET. We found a mean bias of − 0.1 mmHg with limits of agreement of −2.18 to 1.98 mmHg (Tusman et al., 2011a). This confirms the original DuBois’ description of *mean* PACO_2_, resolving the measurement of PACO_2_ at the bedside and allowing the non-invasive calculation of Bohr’s dead space breath by breath.

## Current Interpretation of Bohr’s and Enghoff’s Approaches to Dead Space

According to the previous discussion, it is clear that Bohr’s and Enghoff’s approaches measure different but complementary aspects of gas exchange (Figure 2) (Tusman et al., 2012; Sipmann et al., 2014). The Bohr’s equation measures “true” dead space because it uses parameters exclusively from the alveolar compartment, and thus is based on *the expired alveolar air concept*. The PA-$\bar{\text{E}}$CO_2_ represents the degree of CO_2_ dilution in naturally heterogeneous human lungs. This was confirmed in a model of ARDS where the Bohr’s dead space calculated by VCap was in an excellent agreement (bias 0.01 and LoA −0.04 to 0.06) with the value obtained with the MIGET analysis (Tusman et al., 2011a). The Enghoff’s approach measures not only dead space but also all the spectrum of V/Q mismatch present in the lung (Figure 2). This is because by using PaCO_2_ as a surrogate of PACO_2_ (*the ideal alveolar gas concept*), the effects of low V/Q and shunt are included in its estimation. The true shunt and low V/Q zones let high venous blood PCO_2_ pass through the alveoli increasing PaCO_2_ much above PACO_2_. Some authors called this effect “shunt” dead space (Fletcher et al., 1981), a misleading term as it mixes the two opposite extremes of V/Q mismatch. We observed a poor correlation between the Enghoff’s approach with MIGET dead space (*r* = 0.38; *p* = 0.0078) but a good one with MIGET shunt (*r* = 0.64; *p* < 0.0001) (Tusman et al., 2011a). This is why we think that calling the result of the Enghoff’s equation, a global index of gas exchange, as “dead space” is both physiologically and clinically incorrect!

## Dead Space Subcomponents

The Bohr’s equation calculates the whole *physiological* dead space. It can be expressed as an absolute volume in one breath (VD_phys_ in ml), as part of minute ventilation (VD in L) or, more commonly, as a fraction of VT (VD/VT) (Fletcher et al., 1981; Tusman et al., 2012). VCap is the only clinical monitoring tool that separates the volume of gas within conducting airways from the volume of gas in the alveolar compartment in one breath (Figure 2). The classical geometrical method to identify the midpoint of phase II of the capnogram, described by Fowler (1948), can be replaced by a more accurate mathematical analysis (Tusman et al., 2009). This is of great importance as the slope of phase II represents the emptying of lung units of different time constants and V/Q ratios and the midpoint the averaged interface between convective and diffusive intrapulmonary gas transport. We have named this point as the *airway–alveolar interface* (Figure 1), and it is needed to estimate airway dead space (VD_aw_) and alveolar VT (VT_alv_). Failure to correctly estimate this point can lead to interpretation errors of VCap and dead space components (Tusman et al., 2009). Finally, the alveolar dead space (VD_alv_) is easily obtained by subtracting VD_aw_ from total VD_phys_ (Fletcher et al., 1981). VD_aw_ and VD_alv_ are commonly expressed as a fraction of VT to allow comparisons among different breaths and sizes of patients. The alveolar component is better expressed as a fraction of VT_alv_ (VD_alv_/VT_alv_) because it is an index that closely represents the inefficiency of gas exchange (Fletcher et al., 1981; Tusman et al., 2012).

## Values of Dead Space in Patients With Acute Respiratory Distress Syndrome

Table 1 shows a summary of published Bohr’s and Enghoff’s values from healthy volunteers to patients with ARDS (Nunn and Hill, 1960; Larsson and Severinghaus, 1962; Blanch et al., 1999; Åström et al., 2000; Beydon et al., 2002; Tusman et al., 2013; Doorduin et al., 2016; Gogniat et al., 2018). Mechanical ventilation *per se*, apart from pulmonary diseases, increases dead space. In patients with ARDS, VD is elevated by many other factors including instrumental dead space (HME, elbows, close-suction systems), using low VT ventilation, high respiratory rates, pulmonary vascular involvement, or high positive end-expiratory pressure (PEEP), among others. Instrumental dead space may be of major relevance in patients with ARDS because it is an easily modifiable factor and can sum up to 90 to 100 ml in some unfortunate configurations.

**TABLE 1:** Published values of measured Bohr’s and Enfhoff’s approaches using capnography.

Kind of Patient	Authors	Ventilation	Enghoff’s index	Bohr’s VD/VT	VD_aw_/VT	VD_alv_/VT_alv_
Healthy volunteers	Larsson and Severinghaus (1962) *n* = 11	Spontaneous mean VT ∼ 630 mL	0.23 to 0.31	–	0.18 to 0.24	–
	Åström et al. (2000) *n* = 38	Spontaneous mean VT ∼ 645 mL	Female = 0.23 Male = 0.31	Female = 0.20 Male = 0.26	Female = 0.16 Male = 0.21	–
	Tusman et al. (2013) *n* = 33	Spontaneous mean VT 546 mL	–	0.23 ± 0.08	0.17 ± 0.09	0.07 ± 0.06
Healthy anestetized	Nunn and Hill (1960) *n* = 12	Mandatory mean VT 474 mL	0.32	–	0.13	–
	Tusman et al. (2013) *n* = 33	Mandatory VT 6 mL/kg PEEP 6 cmH_2_O	–	0.28 ± 0.07	0.18 ± 0.08	0.11 ± 0.05
Critically ill anestetized	Unpublished personal data *n* = 55	Mandatory VT 6 mL/kg PEEP 8 cmH_2_O	–	0.41 ± 0.07	0.23 ± 0.07	0.23 ± 0.08
ARDS	Blanch et al. (1999) *n* = 17	Mandatory mean VT ∼ 510 mL PEEP 5–10 cmH_2_O	–	0.53 to 0.63	–	–
	Beydon et al. (2002) *n* = 10	Mandatory mean VT ∼ 625 mL PEEP 5–10 cmH_2_O	0.53 to 0.55	–	0.30 to 0.32	–
	Doorduin et al. (2016) *n* = 15	Mandatory VT 6.8 mL/kg PEEP 12 cmH_2_O	0.68 ± 0.9	0.45 ± 0.7	–	–
	Gogniat et al. (2018) *n* = 14	Mandatory VT 6.5 mL/kg PEEP 10 cmH_2_O	0.70 (0.58–0.74)	0.47 (0.45–0.56)	0.37 (0.31–0.45)	0.19 (0.15–0.23)

*Data is presented as mean ± SD or median (1^st^–3^rd^ quartiles). (–) data not available.*

## Evaluation of Lung Overdistension by Volumetric Capnography

As discussed above, cyclic overdistension is one of the major mechanisms of VILI. It is referred to in different terms depending on whether it denotes functional (hyperdistension) or morphological (hyperinflation) phenomena. Recently, a more conceptual and integrative framework adopted from bioengineering terminology has been introduced to describe the stress or lung deformation that the lung parenchyma suffers during inflation, especially at the end of inspiration when maximal airway pressures are reached. *Lung stress* is defined as the distribution of internal forces per area unit applied to an elastic material, and *lung strain* as the deformation or change in shape of an elastic material from a reference initial value when submitted to a force (Chiumello et al., 2008). Lung parenchyma is constituted by a network of elastic tissues that normally works within a certain range of normal stress and strain. The topographical distribution of stress and strain is heterogeneous due to the natural fractal configuration of the lungs and by the effects of gravity. Lung parenchyma is prone to damage if the normal limits of stress and strain for a particular lung region are exceeded (Protti et al., 2013; Hurtado et al., 2017).

**FIGURE 3:**
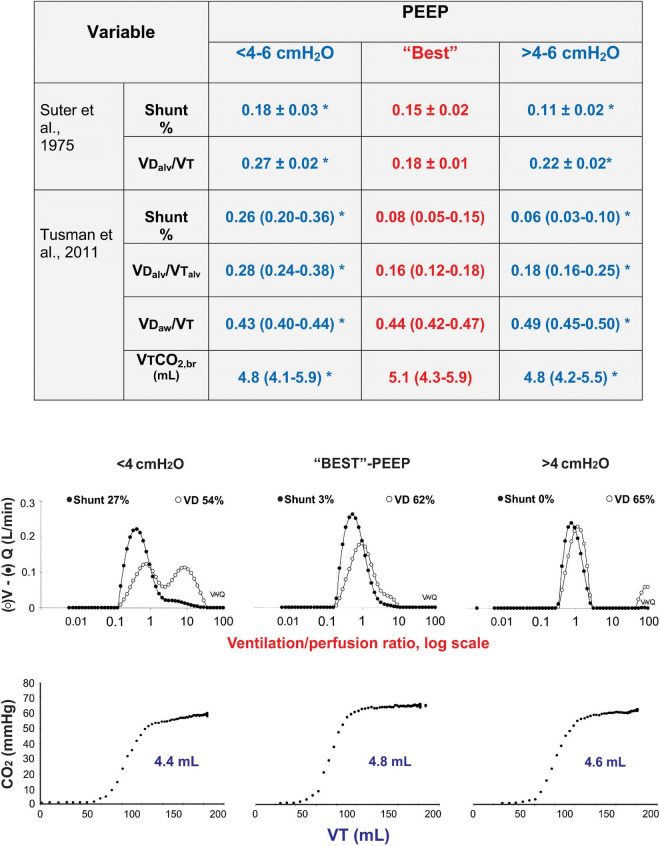
Dead space at optimum PEEP. Comparison of the data obtained by Suter et al. (1975) using Enghoff’s approach with our more recent data using VCap using Bohr’s approach and multiple inert gas elimination technique (MIGET) (Tusman et al., 2011b). Physiological dead space measured by MIGET and VCap airway dead space increase proportional to PEEP, whereas shunt decreases with PEEP. An individualized level of PEEP (“Best” PEEP) corresponding to maximal respiratory system compliance, resulted in the lowest alveolar dead space and the highest elimination of CO_2_ measured by VCap. PEEP above and below this optimum value results in an increased alveolar dead space and decreased the elimination of CO_2_ per breath (VTCO_2,br_). (*) *p* < 0.05 compared to best PEEP.

Volumetric capnography informs about the occurrence of lung overdistension from a different but complementary perspective, as standard and transpulmonary lung mechanics. The Bohr’s dead space increases with positive pressure ventilation, especially in the diseased lungs. The effects of PEEP on VD are well described in the literature (Blanch et al., 1999; Beydon et al., 2002). More importantly, PEEP and the resulting end-inspiratory pressure also increase VD_alv_ as it creates alveolar units with a high V/Q behavior. This is probably the most important parameter related to the risk of developing VILI as it reflects the phenomena occurring at the alveolar level, the most vulnerable part of the lung. Most reports describing the effects of PEEP or MV on VD and VD_alv_ use the Enghoff’s approach, thereby introducing the confounder effect of low V/Q areas on its estimation. In their hallmark paper, Suter et al. (1975) defined the best PEEP as the one resulting in the highest compliance and the lowest VD_alv_. This level also resulted in a reduced shunt, suggesting that the reduction in VD_alv_ could have been responsible in part to this effect. In an animal model of ARDS, we recently reproduced these results by applying 4 cmH_2_O of PEEP below and above the “best” PEEP using the VCap analysis. The Bohr’s derived VD_alv_/VT_alv_ showed the lowest value at the open-lung PEEP and high values with 4 cmH_2_O of PEEP below or above this value (Figure 3) (Tusman et al., 2011b). Of note, by measuring the Bohr’s (i.e., true) dead space, we specifically analyzed the effects of PEEP on the high V/Q component, that is overdistension, eliminating the confounder of shunt or low V/Q regions, which is an important aspect to consider when aiming at monitoring overdistension.

Gogniat et al. (2018) analyzed the effects of PEEP on VD and VD_alv_ in 15 patients with ARDS. They found a similar behavior, i.e., increased overdistension when PEEP was not only above but also below the optimum level set according to the best lung compliance (Table 2) (Gogniat et al., 2018). The authors could identify two clear responses to PEEP when patients were split into two groups according to changes of driving pressure (DP) from baseline ventilation. Patients with DP < 15% (i.e., with better compliance in response to PEEP) presented the lowest dead space and Enghoff’s index values at any PEEP, whereas those with DP > 15% responded exaggeratedly to an increased PEEP. Table 2 shows that the Bohr’s dead space increased proportional to PEEP, whereas Enghoff’s index remained unchanged. As Enghoff’s index includes all V/Q mismatches, it was affected by the opposite simultaneous effects of PEEP on true dead space (increased by overdistension) and shunt (decreased by alveolar recruitment), reducing the sensibility and specificity for detecting lung overdistension.

**TABLE 2:** Dead space and Enghoff’s index in ARDS patients at different levels of PEEP.

Parameters	ΔP	Randomized PEEP (cmH_2_O)			
		
		0	6	10	16
VDBohr/VT	All patients	0.44 (0.41–0.48)	0.45 (0.43–0.52)	0.47 (0.45–0.56)	0.51 (0.46–0.60)
	Δ*P* > 15%	0.50 (0.47–0.54)	0.55 (0.49–0.57)	0.59 (0.51–0.59)	0.61 (0.56–0.65)
	Δ*P* ≤ 15%	0.41 (0.40–0.43)	0.44 (0.42–0.45)	0.45 (0.44–0.46)	0.47 (0.45–0.48)
		*P* = *0.012*	*P* = *0.008*	*P* = *0.006*	*P* = *0.001*
VDaw/VT	All patients	0.33 (0.29–0.36)	0.34 (0.30–0.40)	0.37 (0.31–0.45)	0.39 (0.34–0.47)
	Δ*P* > 15%	0.38 (0.31–0.40)	0.43 (0.33–0.45)	0.48 (0.36–0.50)	0.51 (0.41–0.55)
	Δ*P* ≤ 15%	0.31 (0.29–0.33)	0.31 (0.29–0.34)	0.35 (0.29–0.37)	0.34 (0.30–0.38)
				*P* = *0.047*	*P* = *0.018*
VDalv/VTalv	All patients	0.18 (0.15–0.22)	0.19 (0.17–0.23)	0.19 (0.15–0.23)	0.22 (0.17–0.24)
	Δ*P* > 15%	0.20 (0.19–0.23)	0.22 (0.20–0.24)	0.21 (0.18–0.23)	0.25 (0.24–0.27)
	Δ*P* ≤ 15%	0.16 (0.15–0.24)	0.17 (0.17–0.24)	0.16 (0.13–0.21)	0.16 (0.14–0.21)
			*P* = *0.047*		*P* = *0.008*
Enghoff’s index	All patients	0.71 (0.60–0.73)	0.71 (0.58–0.74)	0.70 (0.63–0.75)	0.69 (0.59–0.77)
	Δ*P* > 15%	0.74 (0.73–0.74)	0.76 (0.74–0.77)	0.76 (0.75–0.76)	0.78 (0.77–0.79)
	Δ*P* ≤ 15%	0.59 (0.56–0.70)	0.58 (0.55–0.69)	0.63 (0.54–0.69)	0.58 (0.53–0.63)
		*P* = *0.025*	*P* = *0.008*	*P* = *0.006*	*P* = *0.002*

*VD_Bohr_/VT = Bohr’s dead space to tidal volume ratio, VD_aw_/VT = airway dead space to tidal volume ratio, VD_alv_/VT_alv_ = alveolar dead space to alveolar tidal volume ratio, and ΔP = driving pressure. Krustal-Wallis non-parametric test for ΔP inter-group comparison. Data is presented as median and 1^st^–3^rd^ quartiles.*

**FIGURE 4:**
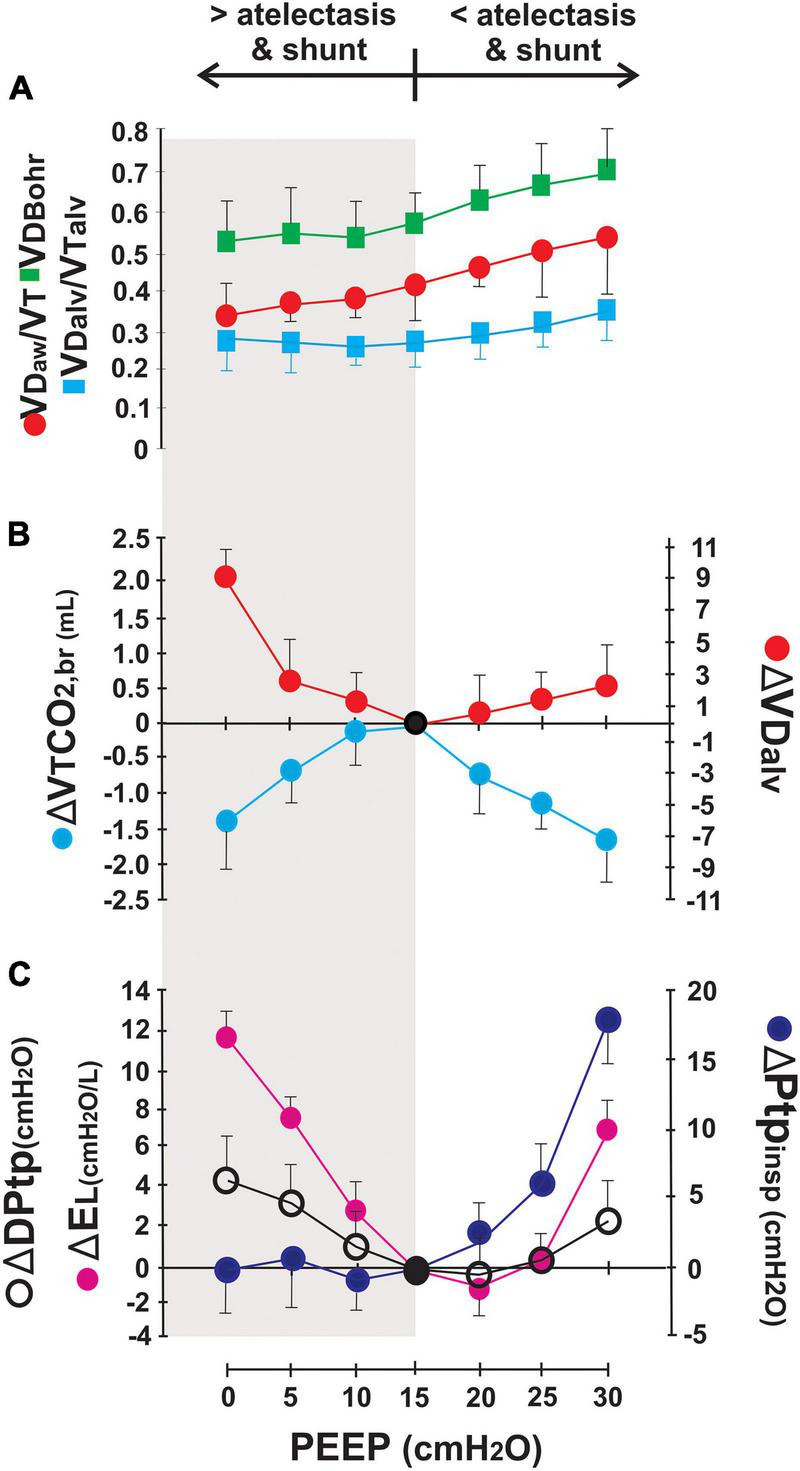
Effects of PEEP in dead space and lung mechanics. Airway dead space increases in proportion to PEEP and has a direct impact on physiological Bohr’s VD. Alveolar dead space (VDalv), on the contrary, changes according to the balance between recruitment and overdistension in the alveolar compartment. Panel **(A)** shows the effect of PEEP on absolute dead space values. Panels **(B,C)** present the differences in alveolar dead space (ΔVD_alv_), elimination of CO_2_ per breath (ΔVTCO_2,br_), transpulmonary driving pressure (ΔDPtp), transpulmonary end-inspiratory pressure (ΔPtp_insp_), and lung elastance (ΔE_L_) between certain PEEP level with the reference15-PEEP considered as the “Best” PEEP according to findings in gas exchange, lung ultrasound, and respiratory mechanics adapted from Tusman et al. (2020).

For examining the role of VD in detecting overdistension in more detail, the effects of increasing PEEP levels from 0 to 30 cmH_2_O were analyzed in an experimental model of ARDS (Tusman et al., 2020). The Bohr’s VD, with both its airway and alveolar components, increased in proportion when PEEP exceeded 15 cmH_2_O, reflecting clear global lung overdistension that was confirmed by a parallel decrease in CO_2_ elimination by the lungs, an increase in lung elastance, transpulmonary DP, and end-inspiratory transpulmonary pressure (Figure 4). However, at PEEP < 10 cmH_2_O, VD_aw_ was minimal but VD_alv_ increased. This increase was associated to a low VTCO_2,br_ and high lung elastance and transpulmonary DP. End-inspiratory transpulmonary pressure remained stable but high (∼20 cmH_2_O). These findings could be interpreted as an increase in stress and strain in the alveolar compartment of the aerated lung, despite lower levels of PEEP, caused by the stressor-raiser role of atelectasis (Figures 4B,C).

## Capnodynamics for Monitoring Lung Strain

Recently, a new method for monitoring lung overdistension by directly measuring lung strain based on expired CO_2_ kinetics, the capnodynamic method, has been described (Suarez-Sipmann et al., 2019). Based on the principles of mass balance for CO_2_ in the lung and the differential Fick principle for CO_2_, the capnodynamic equation provides two highly relevant parameters for monitoring purposes: effective pulmonary blood flow (EPBFCO_2_) (i.e., the non-shunted portion of cardiac output) and end-expiratory lung volume (EELVCO_2_):


$${\text{EELVCO}}_{2}\cdot \left({\text{FACO}}_{2}^{\text{n}}-{\text{FACO}}_{2}^{\text{n}-1}\right)={\text{EPBFCO}}_{2}\cdot \Delta {\text{t}}^{\text{n}}\cdot \left({\text{CvCO}}_{2}-{\text{CcCO}}_{2}^{\text{n}}\right)-{\text{VTCO}}_{2}^{\text{n}}$$

where FACO_2_*^n^* refers to the alveolar fraction of CO_2_ at the *n*th breath and FACO_2_*^n^*^–1^ for the preceding breath. Δ*t^n^* is the duration of the *n*th respiratory cycle, CvCO_2_ and CcCO_2_*^n^* is the mixed venous and capillary CO_2_ content, VTCO_2_*^n^* is the volume of CO_2_ eliminated by the *n*th breath.

The mass balance occurs between the CO_2_ content in the lung (represented on the left side of the equation) equals the difference between the CO_2_ supplied to the lung by perfusion and the amount of CO_2_ eliminated by the lung (on the right side of the equation). To solve the equation for its three unknowns: EELVCO_2_, EPBFCO_2_, and CvCO_2_, a small modification in CO_2_ alveolar concentration must occur under the assumption that CvCO_2_ remains constant during the measurement cycle. All other parameters can be obtained non-invasively by VCap. The change in FACO_2_ is obtained by a minimal modification of the breathing pattern (three consecutive respiratory cycles in which a short expiratory hold is added are repeatedly interspersed between six normal cycles) in a passively breathing patient under MV. Applying a repetitive sequence of six normal and three prolonged breaths, iterative mathematics can solve the capnodynamic equation after a set of nine equations is obtained. From then on, any new breath is added to the sequence providing a new solution (i.e., a new value of EELVCO_2_ and EPBFCO_2_, which can be monitored continuously). A more in-depth description of the method is beyond the scope of this manuscript. The method has been submitted to extensive experimental validation in challenging pulmonary and circulatory conditions (Sander et al., 2014, 2015). Recently, the first validations of EPBFCO_2_ in patients on general anesthesia (Sigmundsson et al., 2021) and of EELVCO_2_ (Öhman et al., 2020) have been published. Both performed well in good agreement with clinical reference methods and excellent trending abilities.

The decrease in FRC is one of the major contributors to VILI during MV. A way to quantify this risk is to determine lung strain, a measure of lung tissue deformation during inflation. To calculate lung strain, VT needs to be normalized to lung volume (strain = VT/FRC) (Chiumello et al., 2008), which during MV is referred to as end-expiratory lung volume as resting volume is influenced by the level of PEEP applied. When a certain threshold of strain is exceeded, the potential for mechanical damage to the ARDS lung increases (Bellani et al., 2011; González-López et al., 2012). The capnodynamic method offers the unprecedented possibility not only to measure EELV at the bedside, which has been very difficult to date, but also to do it in a non-invasive continuous way, extending and complementing the possibilities of VCap to measure lung overdistension and strain.

## Lung Perfusion Estimated Based on Co_2_ Kinetics and Lung Overdistension

The hemodynamic consequence of lung overdistension is crucial information in mechanically ventilated patients with ARDS. Volumetric capnography has the unique ability to describe lung overdistension both from the ventilatory and hemodynamics perspective. Volumetric capnography assesses pulmonary perfusion *qualitatively* through the parameters PETCO_2_ and VCO_2_ (Tusman et al., 2010) and *quantitatively* by calculating the effective capillary pulmonary blood flow (EPBF_CO2_) using equations based on the differential Fick’s formula and the above-described capnodynamic method (Sander et al., 2014, 2015). Lung overdistension induced by high alveolar pressure can collapse pulmonary capillaries, decrease pulmonary blood flow, and increase right ventricle afterload. The association of high dead space with low VCO_2_ or EPBF_CO2_ has been observed during high PEEP ventilation, resulting in a *functional* overdistension because CO_2_ exchange and elimination are impaired.

VCO_2_ or EPBF_CO2_ are decreased not only by lung overdistension but also by other hemodynamical causes like hypovolemia, embolism, arrhythmias, or heart failure. Therefore, physicians involved in the care of ventilated patients should first rule out any other hemodynamic problem when evaluating the hemodynamic consequence of lung overdistension. Again, the context-sensitive nature of CO_2_ kinetics is relevant to make differential diagnoses. Many questions arise when the operator observes changes in pulmonary perfusion in ventilated patients. Is any acute hemodynamic problem of extra-pulmonary origin responsible for the low elimination of CO_2_? Is the patient with normovolemia or has a preload-dependency? Have alveolar ventilation or body metabolism changed?

## Prediction of Acute Respiratory Distress Syndrome Outcome

The role of VCap to determine the prognosis of patients with ARDS has been well established. Enghoff’s index has been found to be strongly associated with mortality in the early and late course of ARDS (Nuckton et al., 2002; Kallet et al., 2014). The same research group showed that the risk to death increased by 22% for every 0.05 increase in Enghoff’s index (OR = 1.22, 95% CI 1.11–1.35, *p* < 0.001) in patients with moderate and severe ARDS (Kallet et al., 2017). The magnitude of changes in the Enghoff’s index varied according to ARDS etiology although, in each ARDS subgroup, this variable was always higher in non-survivors than in survivors. Recently, surrogates of VD, such as the ventilatory ratio—an index calculated by the quotient between measured and predicted minute ventilation and PaCO_2_—was independently associated with mortality in patients with ARDS (Sinha et al., 2019). Why are these CO_2_-based indexes, such good predictors of survival, better than the usual oxygen-based index in ARDS? When PaCO_2_ is used to calculate dead space (according to the ideal alveolar gas concept) what it is measured is a global index of gas exchange including low V/Q and shunt. Lung physiology explains that shunt is represented not only by the PA-aO_2_ but also by the Pa-ACO_2_ difference. Therefore, these indexes based on PaCO_2_ reflect the severity of ARDS by assessing shunt in combination with dead space. The prognostic value of Bohr’s “true” dead space in a patient with ARDS is still unknown, but it is very likely that it also has an important role as a direct measure of lung ventilatory inefficiency and overdistension, both with likely strong influence on outcome.

## Conclusion

Analysis of expired CO_2_ kinetics by VCap provides important non-invasive cardiorespiratory information for clinical assessment, monitoring, and management of ARDS mechanically ventilated patients. Dead space and the Enghoff’s index are calculated with high precision even in patients with very severe lung injury, as those with ARDS. The concept of VD is clinically useful not only to assess and adjust alveolar ventilation in the context of lung-protective MV but also to detect alveolar overdistension. Capnodynamic measurement of end-expiratory lung volume allows estimating strain that in combination with lung mechanics and lung perfusion can provide a more in-depth understanding of the lung condition and detect when this value exceeds safe limits. Real-time assessment of the elimination of CO_2_ and effective pulmonary capillary blood flow provides information about the hemodynamic consequences of positive pressure ventilation. The combination of an increase in VD and a decrease in pulmonary capillary blood flow characterizes a situation of *functional* lung overdistension. Further studies are needed to explore the precise cutoff value to define harmful functional overdistension with VD and to determine its role as a screening tool to predict the evolution in patients with ARDS.

## Author Contributions

All authors listed have made a substantial, direct, and intellectual contribution to the work, and approved it for publication.

## Conflict of Interest

The authors declare that the research was conducted in the absence of any commercial or financial relationships that could be construed as a potential conflict of interest.

## Publisher’s Note

All claims expressed in this article are solely those of the authors and do not necessarily represent those of their affiliated organizations, or those of the publisher, the editors and the reviewers. Any product that may be evaluated in this article, or claim that may be made by its manufacturer, is not guaranteed or endorsed by the publisher.
